# Society Proceedings

**Published:** 1902-12

**Authors:** 


### SOCIETY PROCEEDINGS.

### PENNSYLVANIA STATE VETERINARY MEDICAL ASSOCIATION.

Resolutions adopted at the semi-annual meeting of the Pennsylvania State Veterinary Medical Association meeting, Reading, Pa., September 16, 1902.

### REGISTRATION.

WHEREAS, The need of more adequate laws for the regulation of licensed practitioners of Pennsylvania is needed in order that the several laws regulating the same may be more rigidly enforced; therefore be it

*Resolved,* That we, the Pennsylvania State Veterinary Medical Association in convention assembled, do most earnestly recommend that our State Legislative Committee be instructed to draft a suitable bill for presentation at the coming session of the Legislature, requiring biennial registration of all licensed practitioners; and be it further

*Resolved,* That we recommend that all such registrations shall be through the State Board of Veterinary Medical Examiners.

### D. C. STANTON.

WHEREAS, It has pleased Almighty God to remove from our midst our late colleague, Dr. D. C. Stanton; and

WHEREAS, His association with our organization was marked by a deep and continued interest in all our work, the faithful discharge of every duty to his profession; therefore be it

*Resolved,* That we deeply mourn his loss and tender to his family our heartfelt condolence in their and our great loss; and be it further

*Resolved,* That a copy of these resolutions be spread upon our minutes, and also sent to the family of the deceased.

### LOCAL COMMITTEE OF ARRANGEMENTS.

WHEREAS, Our semi-annual meeting at Reading has been marked with an unusual attendance and the most widely representative gathering of the profession at any of its semi-annual meetings for the past ten years; and

WHEREAS, The activity of the veterinary profession of the Schuylkill Valley and the members of the profession in this city has given impetus to this splendid gathering; and

WHEREAS, Our entertainment in the city of Reading has been of so pleasant and enjoyable a character; therefore, be it

*Resolved*, That we extend to the Schuylkill Valley Association and Local Committee of Arrangements our grateful acknowledgment and thankful appreciation of their many courtesies.

W. HORACE HOSKINS,
Chairman Committee on Resolutions.

---

### PASSAIC COUNTY VETERINARY MEDICAL ASSOCIATION.

THE regular monthly meeting of the Association was held at 169 Paterson Street, Paterson, N. J., on Tuesday evening, November 4, 1902, with Dr. William Herbert Lowe, President, in the chair, and Dr. Alexander Machan, Secretary.

Notwithstanding the fact that it was election night there was a quorum in attendance, the following practitioners being present:

Drs. T. J. Cooper, David Machan, Alexander Machan, John H. DeGraw, W. H. Lowe, Jr., J. Payne Lowe, Passaic; William Herbert Lowe, Paterson.

The minutes of meeting of October 7 were read and approved.

Under "unfinished business" the President reported that he had ordered certificates of membership, which would be ready in the course of a few days.

Secretary A. Machan reported that he had up to date made two payments to Treasurer M. A. Pierce, the first amount being $16, and the second $10, making a total of $26 paid over to the Treasurer. The Secretary further reported that he had $2 in hand just paid him by one of the members, and that all members had paid their dues for this year except four who were not present when he was collecting dues.

Dr. Machan also reported that he had sent invitations to Bergen county practitioners, as instructed by the Association at the last meeting.

The Secretary's report was received and ordered entered upon the minutes.

Treasurer M. A. Pierce not being present, there was no report from that officer.

President Lowe announced that he had had some correspondence with the State Board of Health as to whether veterinarians could have the use of the State Bacteriological Laboratory at Trenton for diagnostic purposes, and he was pleased to be able to give a favorable report, and he hoped that veterinarians would avail themselves of the services of the State Laboratory. Dr. Lowe stated that the work at the State Laboratory is conducted free of charge, and that it consists in examinations for diagnosis in the various affections which are produced by micro-organisms. Communicable diseases of whatever character are investigated, and a diagnosis is made when possible. Investigations of a private nature will not be undertaken at the laboratory, and veterinarians are requested not to send sections of tumors, etc., to the laboratory, as no examinations will be made of such substances, the work of the laboratory being wholly devoted to the public health interests of the State.

Inquiries will be made into the character and purity of the animal products in use in this State for prophylactic and remedial purposes, and also concerning the causes of wholesale poisoning due to unwholesome food, and into the germicidal value of the various substances employed in sanitary operations.

Specimens of tissues suspected to be affected with rabies, anthrax, glanders, and other diseases peculiar to the lower animals can be forwarded in the manner described in Circular 105 of the State Board of Health, issued August, 1902. The ordinary mailing-cases for the other diseases named in the said circular can be obtained in Paterson at the office of the Board of Health or at the drug store of Gurdon E. Pellett, corner of Park avenue and Carroll street. Dr. Lowe mentioned that Dr. Cooper had already availed himself of the use of the State Laboratory by taking a portion of the brain of a horse supposed to have rabies to Trenton, for diagnostic purposes, and Dr. Cooper explained why in this case he expected the result would be negative.

The President stated that he had not been able to attend the annual meeting of the New Jersey Sanitary Association at Lakewood, October 24 and 25, and make the report of the Committee on Animal Diseases and Animal Foods of that organization, of which committee he is chairman.

Dr. Cooper brought up his black-list proposition again, and, on motion of Dr. Alexander Machan, it was laid over until the next meeting.

Dr. Pope telephoned his regrets at not being able to be present.

Dr. William C. Berry formerly of Bloomingdale, now of Haskell, wrote a letter showing his deep interest in the Association.

Dr. Cooper, who had been appointed essayist for the meeting, read an excellent paper on "Milk as a Conveyer of Disease." The subject being an important one, it was fully discussed. Dr. Machan moved that a vote of thanks be given Dr. Cooper, and that his paper be sent to the *Review* and to the JOURNAL for publication. Carried.

The President appointed Dr. David Machan essayist for the December meeting, subject, "Examination of Horses for Soundness."

On motion, meeting adjourned at 10 P.M.

A. MACHAN,
Secretary.

---

### ALLEGHENY COUNTY VETERINARY MEDICAL ASSOCIATION.

A VERY interesting and instructive meeting was held at Dr. J. E. Spindler's office on the evening of November 12th. Members present: Drs. Birch, Boyd, Emery, Hinman, Spindler, Spohn, Taylor, and Waugh. Visitors—Drs. A. T. Roll, of Natrona, and Wm. J. Waugh, of Washington. Routine business was transacted, and committees were appointed for future work, and plans formulated to enlarge the Association and increase its sphere of usefulness, then occasionally hold surgical clinics.

Dr. James A. Waugh exhibited Dr. N. Rectenwald's new steel clamp for removing collar tumors and shoe boils; explained its application and uses, and showed a large shoe boil as removed that day in his practice. Dr. A. W. Hinman made some practical remarks on observation or subnormal temperatures in animals. Hair balls in calves' stomachs proved an interesting subject when Dr. James A. Waugh presented seventeen different-sized and multicolored hair balls, received from his friend, Dr. Benj. Howse, U. S. Vet. Inspector in Allegheny. Dr. John E. Spindler gave an original and unique lecture, entitled "Greasing a Mit," which dealt freely and fully with the baneful practice of tipping coachmen and whacking-up with stable-bosses. Dr. James A. Waugh mentioned an improvement in the surgical technique of Professor C. C. Lyford's operation for the radical cure of bursal enlargements. There was free and friendly discussion of all those subjects presented for consideration. Members are exchanging text-books, and manifest much interest in advancement, and a vigorous professional spirit prevails.

JAMES A. WAUGH, V. S.,

Secretary.

---

### ILLINOIS STATE VETERINARY MEDICAL ASSOCIATION.

THIS Association held its twentieth annual meeting in Exchange Hall, Union Stock Yards, Chicago, Ill., Tuesday and Wednesday, December 2 and 3, 1902. Meeting was called to order by the President, Dr. Joseph Hughes, at 10.30 A.M. Minutes of previous meeting read and approved. The society then listened to the President's annual address.

The following members and visitors were in attendance during the meeting: Drs. R. G. Walker, Joseph Hughes, D. S. Jaffray, Jr., C. F. Greinier, J. F. Ryan, E. L. Quitman, James Robertson, G. A. Lytle, L. A. Merillat, A. H. Baker, Robert Gysell, F. W. Buecher, Chicago; N. I. Stringer, Watseka; L. C. Tiffany, Albert Babb, Springfield; J. F. Pease, Quincy; C. C. Mills, Decatur; W. F. Scott, Oak Park: J. S. Hollingsworth, La Salle; W. B. Lewin, Russell; F. H. Barr, Pana; W. H. Curtis, Marengo; R. W. Story, Princeton; F. W. Kee, Sheldon; W. C. Hannawalt, Sheffield; G. B. Jones, Sidell; H. A. Pressler, Fairbury; J. M. Kaylor, Barry; C. D. Maulfair, McNabb: W. J. Martin, Kankakee; W. C. Galbraith, Wheaton; F. B. Rowan, Belvidere; J. H. Crawford, Harvard; E. F. Beckley, Rockford; James Smellie, Eureka; C. P. Draper, Arlington Heights; W. C. Galbraith, Wheaton; J. T. Nattress, Delavan; W. H. Welch, Lexington; R. F. Hoadley, Yorkville; Thomas P. Brankin, Joliet; C. H. Merrick, Okawville; J. S. Spangler, Aurora. Visitors—Dr. Davis, H. W. Hawley, O. E. Dyson, Dr. Johnson, Dr. Redmon, Dr. Frost, Chicago; S. E. Gates, Blandinsville; also Dr. Brimhall, of the State Experiment Station of Minnesota.

The following applications for membership were received, and on motion the applicants were duly elected: Dr. Wm. W. Welch (Ontario), Elgin; W. C. Galbraith (Ontario), Wheaton; Thomas P. Brankin (McKillip), Joliet; Chauncey D. Maulfair (Chicago), McNabb; E. F. Beckley (Chicago), Rockford; J. S. Hollingsworth (Ontario), La Salle; W. H. Curtiss (Chicago), Marengo; C. S. Hayward (Ontario), Mattoon; J. M. Kaylor

(Chicago), Barry; F. N. Godsall (Chicago), Chicago; C. P. Draper (McKillip). They were recommended by Quitman, Stringer, Gysel, Robertson, Lewin, Martin, Smellie, Pease, and Babb.

Auditing Committee reported Treasurer's books O. K. Treasurer's report showed balance on hand, $83.69. Bills for printing and Secretary's fees were allowed to the amount of $23.65.

Motion carried that Secretary have five hundred copies of Constitution and By-laws printed as revised.

Society proceeded to elect officers for ensuing year, resulting as follows: President, Dr. H. A. Pressler, Fairbury; Vice-President, Dr. N. I. Stringer, Watseka: Secretary, Dr. W. H. Welch, Lexington; Treasurer, Dr. R. G. Walker, Chicago; Board of Censors, Dr. J. F. Pease, Quincy; Dr. A. H. Baker, Chicago; Dr. A. C. Worms, Chicago.

A vote of thanks was tendered Drs. Baker and Hughes for their hospitality.

Dr. Pressler then took the chair and appointed the following committees: Programme—Dr Albert Babb, Springfield; Dr. W. F. Kee, Sheldon; Dr. F. J. Ryan, Chicago. Arrangements—Dr. C H. Merrick, Okawville; J. T. Nattress, Kankakee. Legislation—Dr. E. L. Quitman, Chicago; Dr. James Robertson, Chicago; Dr. W. J. Martin, Kankakee.

Dr. Beckley favored the society with a song.

Adjourned to meet at Champaign, in February, at the call of the President.

W. H. Welch,
Secretary.

---

### EXPOSITION OF HYGIENIC MILK SUPPLY.

The United States Department of Agriculture has received through the Department of State notice that a general exposition of hygienic milk supply will be held at Hamburg from May 2 to May 10, 1903. The exposition will embrace eight sections as follows:

Section A.—For milk production: (1) Exhibit of limited number of milch cows of known race; (2) stable fittings and implements; (3) regimen and hygienic food; (4) technics of milk, tests, and execution of; (5) management of milk in stable and pastures; (6) personnel of milking and stable (clothing, health, and supervision of the same).

Sec. B.—Veterinary control of the condition of milch cows and of milk; (1) Legislation; (2) management of contagious outbreaks (with demonstration); (3) diseases of milch cows; (4) special diseases; (5) unwholesome food plants and drinking water; (6) secretion through the milk of medicinal stuffs; (7) sanitary management; (8) disinfection of stalls (means and apparatus).

Sec. C.—Conveyance of milk, land, and waterways, railways; conveyance and distribution in cities; (2) cleansing, spinning, cooling, Pasteurizing, sterilizing, and concentrating (condensing) milk; (3) arrangements for measuring and weighing; (4) cleansing apparatus for flasks; (5) machinery for bottling, pouring, and sealing.

SEC. D.—Exhibit of management and sale of milk (wholesale and retail trade), with complete furnishings.

SEC. E.—Milk legislation and administration: (1) Laws, ordinances, decrees, and judgments; (2) police supervision of milk traffic (removal, previous examination, preserving, conveyance); (3) chemical and bacteriological inspection; (*a*) model laboratory, working; (*b*) instruments and tools for laboratory.

SEC. F.—Scientific: (1) Means of instruction, with scientific demonstration; (2) scientific instruments and tools for milk laboratories; (8) literature, statistics, and graphic exhibitions.

SEC. G.—Milk preparations: (1) Condensed and prepared for long keeping for use in the army and navy; (2) milk for infants; (3) for therapeutic purposes; (4) other foods and preparations produced from milk.

SEC. H.—Machinery and apparatus for the treatment of milk in the household.

The cost for space for exhibits on the main floor will be $2.38 per square meter (1.196 square yards); side rooms, $1.19, and in the open air, 71 cents. An additional charge of 10 to 50 per cent. will be made for sites with four sides exposed, or with three sides exposed, from 5 to 25 per cent.

Intending exhibitors should make application for space to the Geschaftstelle in Hamburg, 6, Kamp Strasse 46.

Articles for exhibition must be sent and removed at the expense and risk of the exhibitor.

---

### ASSOCIATION MEETINGS.

The Schuylkill Valley Veterinary Medical Association will hold their semi-annual meeting at Reading, Pa., December 17, 1902. Papers will be presented by Dr. Leonard Pearson, Philadelphia, Pa., on "The Veterinary Teacher and Practitioner;" Dr. S. G. Burkholder, Rothsville, Pa., "An Historical Essay, Relation of the Veterinarian to the Medical Profession;" Dr. E. M. Ranck, Glenolden, Pa., "Action of Sera in the Blood;" Dr. D. E. Kohler, Boyertown, Pa., "Reports of Cases;" Dr. G. A. Wehr, Denver, Pa., "Pleuropneumonia."

The annual meeting of the Veterinary Medical Association of New Jersey, at Trenton, in January, is sure to attract a very large attendance, and the programme promised fully warrants the outlook.

THE Illinois Veterinary Medical and Surgical Association will hold their fourteenth annual meeting at the St. Nicholas Hotel, Decatur, Illinois, January 14 and 15, 1903. The programme will include papers by Dr. W. J. Martin, Kankakee, "Veterinary Dentistry;" Dr. J. M. Reed, Matoon, "Retained Fœtal Envelopes;" Dr. C. A. Hurlbutt, Stonington, "Esophageal Obstruction;" Dr. S. H. Swain, Decatur, "Persistency of the Urachus;" Dr. V. G. Hunt, Arcola, "Prolapsus Uteri;" Dr. John Osborne, Nokomis, "Vegetable Poisoning, Milk Sickness;" Dr. N. P. Whitmore, "Hypodermic Stimulation;" Dr. F. Glassbrenner, North Alton, "Foot Lameness;" Dr. D. K. Goodale, Mt. Vernon, "Osteoporosis;" Dr. J. W.

Marsh, Illiopolis, "Parasitic Bronchitis;" Dr. R. W. Braithwaite, Champaign, "Horseshoeing;" Dr. I. M. Luzader, Nokomis, "Caponizing," besides other reports of cases.

---

The students of the San Francisco Veterinary College have organized and will maintain a Veterinary Medical Association in connection with their college work.

